# γδ T Cells Are Required for Pulmonary IL-17A Expression after Ozone Exposure in Mice: Role of TNFα

**DOI:** 10.1371/journal.pone.0097707

**Published:** 2014-05-13

**Authors:** Joel A. Mathews, Alison S. Williams, Jeffrey D. Brand, Allison P. Wurmbrand, Lucas Chen, Fernanda MC. Ninin, Huiqing Si, David I. Kasahara, Stephanie A. Shore

**Affiliations:** Molecular and Integrative Physiological Sciences Program, Department of Environmental Health, Harvard School of Public Health, Boston, Massachusetts, United States of America; University of Colorado, Denver, United States of America

## Abstract

Ozone is an air pollutant that causes pulmonary symptoms. In mice, ozone exposure causes pulmonary injury and increases bronchoalveolar lavage macrophages and neutrophils. We have shown that IL-17A is important in the recruitment of neutrophils after subacute ozone exposure (0.3 ppm for 24–72 h). We hypothesized that γδ T cells are the main producers of IL-17A after subacute ozone. To explore this hypothesis we exposed wildtype mice and mice deficient in γδ T cells (TCRδ^−/−^) to ozone or room air. Ozone-induced increases in BAL macrophages and neutrophils were attenuated in TCRδ^−/−^ mice. Ozone increased the number of γδ T cells in the lungs and increased pulmonary *Il17a* mRNA expression and the number of IL-17A^+^ CD45^+^ cells in the lungs and these effects were abolished in TCRδ^−/−^ mice. Ozone-induced increases in factors downstream of IL-17A signaling, including G-CSF, IL-6, IP-10 and KC were also decreased in TCRδ^−/−^ versus wildtype mice. Neutralization of IL-17A during ozone exposure in wildtype mice mimicked the effects of γδ T cell deficiency. TNFR2 deficiency and etanercept, a TNFα antagonist, also reduced ozone-induced increases in *Il17a* mRNA, IL-17A^+^ CD45^+^ cells and BAL G-CSF as well as BAL neutrophils. TNFR2 deficient mice also had decreased ozone-induced increases in Ccl20, a chemoattractant for IL-17A^+^ γδ T cells. *Il17a* mRNA and IL-17A^+^ γδ T cells were also lower in obese *Cpe^fat^* versus lean WT mice exposed to subacute ozone, consistent with the reduced neutrophil recruitment observed in the obese mice. Taken together, our data indicate that pulmonary inflammation induced by subacute ozone requires γδ T cells and TNFα-dependent recruitment of IL-17A^+^ γδ T cells to the lung.

## Introduction

γδ T cells are a key component of the innate immune response, especially at mucosal surfaces. These cells are found throughout the lung, particularly in the subepithelial region, where they may regulate other immune cells including macrophages and dendritic cells [1]. γδ T cells are an important source of IL-17A, a key cytokine involved in neutrophilic inflammation [2]. In mice, the number of pulmonary γδ T cells increases following infection with certain bacteria [3]. Mice deficient in γδ T cells (TCRδ^−/−^ mice) have attenuated pulmonary clearance of these bacteria, likely as a result of loss of IL-17A production by γδ T cells and consequent reduced neutrophil recruitment [4]. The number of γδ T cells in the lung also increases under conditions associated with oxidative stress, including smoking, bleomycin instillation, and allergen challenge [5]–[8]. Moreover, the pulmonary inflammation induced by such agents requires γδ T cells.

Inhalation of ozone (O_3_), a common air pollutant, has a significant impact on human health. O_3_ causes respiratory symptoms and reductions in lung function [9]–[13]. O_3_ also increases the risk of respiratory infections and is a trigger for asthma [14]–[16]. Exposure to O_3_ induces oxidative stress in the lung, damages lung epithelial cells, and causes the release of numerous cytokines and chemokines that recruit neutrophils and macrophages to the lung [9], [17]. We have reported increased *Il17a* mRNA expression and increased numbers of IL-17A^+^ γδ T cells in the lungs after subacute O_3_ exposure (0.3 ppm O_3_ for 24–72 h) [18]. Hence, we tested the hypothesis that γδ T cells, via their ability to produce IL-17A, are involved in orchestrating the inflammatory response to subacute O_3_ exposure. We examined IL-17A expression in WT and TCRδ^−/−^ mice after exposure to air or to O_3_ (0.3 ppm for 24–72 h). We also examined the effect of IL-17A neutralizing antibodies on O_3_-induced inflammation. Our results indicate an important role for IL-17A^+^ γδ T cells in the inflammatory cell recruitment induced by subacute O_3_ exposure.

TNFα a pleiotropic pro-inflammatory cytokine, enhances the recruitment of neutrophils to the lungs in response to a variety of noxious stimuli, including LPS [19], cigarette smoke [20], and enterobacteria [21]. TNFαis also required for neutrophil recruitment after subacute O_3_ exposure [22], [23]. However, TNFα does not have direct chemoattractant activity for neutrophils [24]. Instead, TNFα recruits neutrophils in part by inducing expression of other cytokines and chemokines [24], [25]. In several pathological states, TNFα induces the expression of IL-17A [26], [27]. Hence, we hypothesized that TNFα contributes to neutrophil recruitment following subacute O_3_ exposure by promoting recruitment to or activation of IL-17A^+^ γδ T cells in the lungs. We used two methods to test this hypothesis. First, we assessed the effect of O_3_ exposure on pulmonary *Il17a* expression and recruitment of IL-17A^+^ γδ T cells in WT mice and in mice deficient in TNFR2 (TNFR2^−/−^ mice). Others have established that either TNFR1 or TNFR2 deficiency reduces the inflammatory response to subacute O_3_, and there is no further impact of combined TNFR1/TNFR2 deficiency [22]. Second, we examined the impact of the TNFα antagonist, etanercept, on *Il17a* expression. Our data suggest that TNFα is required for the recruitment of IL-17A^+^ γδ T cells to the lung after subacute O_3_ exposure.

Approximately one third of the US population is obese and another third is overweight, but our understanding of how obesity impacts pulmonary responses to O_3_ is still rudimentary. Such an understanding may have broad reaching implications since oxidative stress also contributes to responses to a variety of other noxious stimuli [5]–[8], many of which are affected by obesity [28], [29]. In mice, the impact of obesity on responses to O_3_ depends on the nature of the exposure: the pulmonary inflammation induced by acute O_3_ exposure (2 ppm for 3 h) is augmented in all types of obese mice examined to date [30]–[33], whereas the pulmonary inflammation induced by subacute O_3_ exposure (0.3 ppm for 24–72 h) is reduced [34]. Given our findings of the requirement for TNFα-recruitment of IL-17A producing γδ T cells in the induction of pulmonary inflammation after subacute O_3,_ we sought to determine if changes in the activation of γδ T cells might explain the reduced responses to subacute O_3_ we observed in obese *Cpe(*carboxypeptidase E*)^fat^* mice. Data described below indicate that the reduced O_3_-induced neutrophil recruitment observed in obese mice is likely the result of reduced *Il23* expression leading to reduced IL-17A^+^ γδ T cells. Given the importance of IL-17^+^ γδ T cells for responses to viral and bacterial pathogens (see above), these observations might explain the altered response of the obese to bacteria and virus (see review by Peter Mancuso [35]).

## Methods

### Animals

This study was approved by the Harvard Medical Area Standing Committee on Animals. Male age-matched WT and TCRδ^−/−^ mice were either purchased from The Jackson Laboratory (Bar Harbor, ME) and acclimated for 4 weeks, or bred in house. *Cpe^fat^* mice are deficient in carboxypeptidase E, an enzyme involved in processing neuropeptides involved in eating behaviors [36]. The breeding strategy used to generate *Cpe^fat^/*TNFR2^−/−^ mice from *Cpe^fat^* and TNFR2^−/−^ mice (also originally purchased from The Jackson Laboratory) was previously described [37]. All mice were on a C57BL/6J background, fed a standard mouse chow diet, and were 10–13 weeks old at the time of study.

### Protocol

For comparisons of WT and TCRδ^−/−^ mice, mice were exposed to O_3_ (0.3 ppm) or to air, for 24–72 hours, as previously described [18]. Mice were exposed in normal cages without the microisolator top, but with free access to water and food throughout exposure. Mice were checked daily. At least two mice were placed in each cage to limit stress. After exposure, mice were euthanized with an overdose of sodium pentobarbital. The trachea was cannulated and bronchoalveolar lavage (BAL) was performed. After BAL, the lungs were flushed of blood by injecting 10 ml of cold PBS through the right ventricle, after creating a large excision in the left ventricle. One lung was excised and used for flow cytometry. The other was excised and placed in RNAlater (Qiagen, Germantown, MD) for preparation of RNA for real time PCR. In another cohort, WT mice were injected i.p. with 100 µg of anti–IL-17A neutralizing monoclonal antibody (Ab) (Rat IgG2A, clone 50104, MAB421; R&D Systems, Minneapolis, MN) or isotype control Ab (clone 54447, MAB006; R&D Systems) in 100 µl of sterile saline 24 hours before O_3_ exposure. Mice were exposed to O_3_ for 72 hours, euthanized, and tissues were harvested as described above. In a separate series of experiments, WT, TNFR2^−/−^, *Cpe^fat^*, and *Cpe^fat^*/TNFR2^−/−^ mice were exposed to room air or O_3_ (0.3 ppm) for 48 h followed by BAL and tissue harvest. In other experiments, WT and *Cpe^fat^* mice were treated twice (48 h and 1 h prior to O_3_ exposure) with the TNFα blocking drug, etanercept (30 mg/kg s.c.) (Immunex, Thousand Oaks, CA), or vehicle. A similar etanercept dosing regimen has been shown to be effective in inhibiting TNFα in mice over the time course of O_3_ exposures we used (48 h) [38], [39].

### Bronchoalveolar Lavage

BAL was performed and cells counted as previously described [18]. BAL supernatant was stored at −80°C until assayed. BAL KC, IL-6, MCP-1, IP-10 and G-CSF were measured by ELISA (R&D Systems). In mice treated with anti-IL-17A, BAL cytokines and chemokines were measured by multiplex assay (Eve Technologies, Calgary, Alberta). Total BAL protein was measured by Bradford assay (Bio-Rad, Hercules, CA).

### Flow Cytometry

Left lungs were harvested and placed on ice in RPMI 1640 media containing 2% FBS and HEPES. Lungs were digested and prepared for flow cytometry as previously described [18]. Cells were stained using the following antibodies: Alexa Fluor 647 anti-IL-17A (clone: TC11-18H10.1), PE anti-TCRδ (clone: GL3), PE-cy7 anti-CD45 (clone: 30-F11), and APC-cy7 anti-CD3 (clone: 17A2) (all antibodies from Biolegend). Isotype control antibodies were used to set all gates. Cells were visualized using a Canto II (BD Biosciences) and the data was analyzed using Flowjo (Tree Star; Ashland, OR).

To determine if TNFα impacted IL-12Rβ1 expression on lung γδ T cells, lungs from WT mice were digested as above and then cultured in complete RPMI media (RPMI 1640 (Corning, Tewksbury, MA), 10% FBS (Life Technologies), 2 Μm L-glutamine (Life Technologies), 100 units/ml Pen/Strep (Lonza, Hopkinton, MA) and 20 Μm Hepes (Thermo Scientific, Tewksbury, MA)). Cells were plated at a concentration of 10^6^ cells/ml in 24 well plates with or without 100 ng/ml of recombinant murine TNFα (R&D Systems) [40]. Cells were harvested after 24 h, washed with PBS, and stained using the following antibodies: anti-CD16/32 (True Stain biolegend), Strep-APC (Biolegend), PE anti-CD212 (IL-12Rβ1) (BD Biosciences), Biotin anti-TCRδ (clone: GL3, biolegend), PE-cy7 anti-CD45 (clone: 30-F11) and analyzed by flow cytometry as described above.

### Real-time PCR

RNA was extracted from lung tissue and prepared for qPCR using the SYBR method as previously described [18]. All expression values were normalized to 36B4 expression using the ΔΔCt method. The primers for *Il17a* and *36B4* were previously described [37]. Primers for *Ccl20*, *Il23* (p19) and *Il12Rβ1* are described in Table 1. For each set of primers, melt curve analysis yielded a single peak. *Il12Rβ1* expression was measured at baseline in order to tease apart the effects of genotype (deficiency of TNFα signaling versus sufficient signaling) versus O_3_ exposure.

**Table 1:** Primers used for real time PCR.

*Il23p19*	F: CCC ATG GAG CAA CTT CAC AC R: GCT GCC ACT GCT GAC TAG AAC
*Ccl20*	F: AAG ACA GAT GGC CGA TGA AG R: AGG TTC ACA GCC CTT TTC AC
*Il12Rb1*	F: GTG CTC GCC AAA ACT CGT TT R: GGA TGT CAT GTT GCC TCC CA

### Statistical Analysis

Data were analyzed by factorial ANOVA using STATISTICA software (Statistica, StatSoft; Tulsa, OK) with mouse genotype and exposure as main effects. Fisher’s least significant difference test was used as a post-hoc test. BAL cells and flow cytometry data were normalized by log transformed prior to analysis. A *p* value <0.05 was considered significant.

## Results

### O_3_-induced Inflammation is Reduced in TCRδ^−/−^ Mice

**Figure 1:**
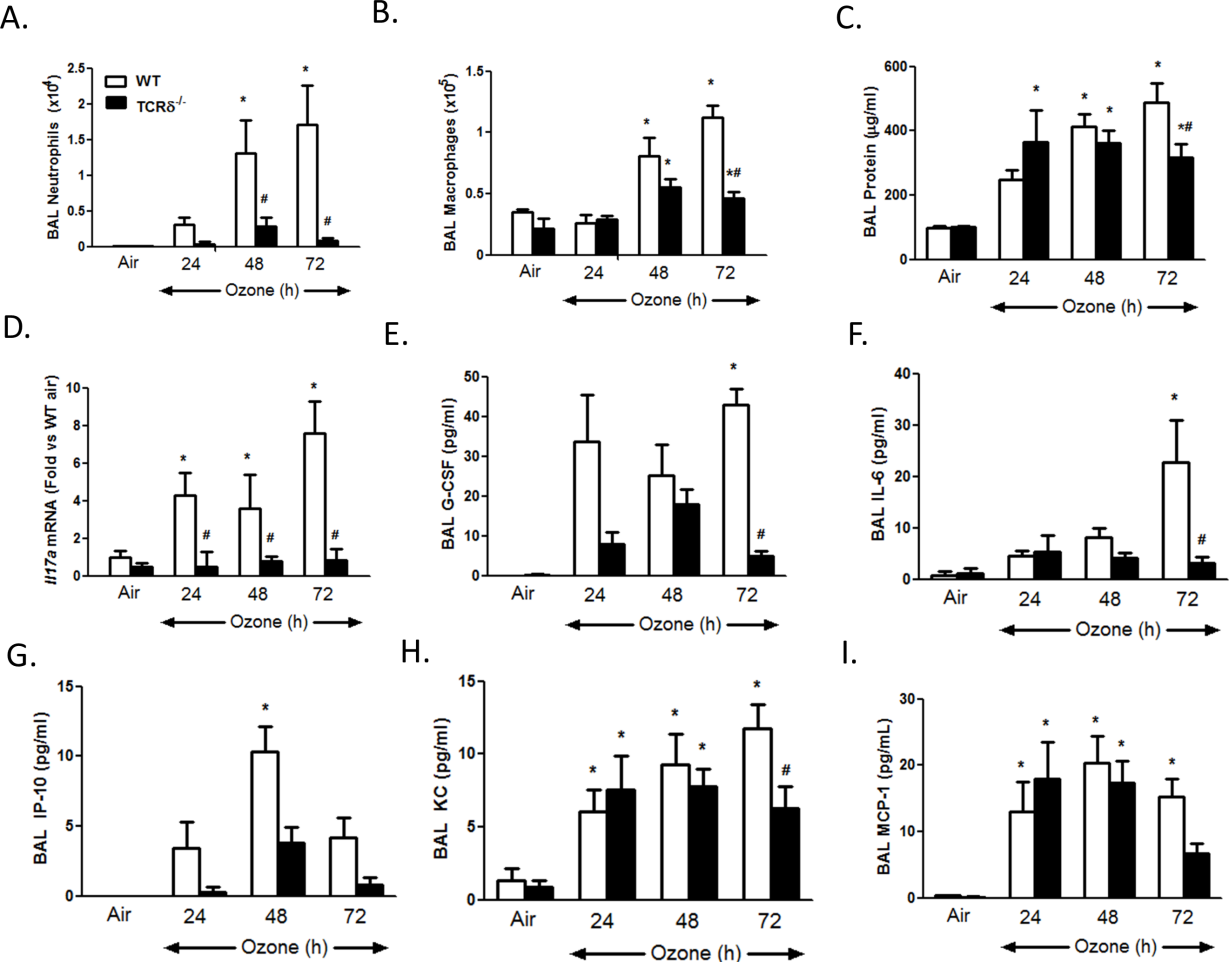
Effect of γδ T cell deficiency on pulmonary inflammation and injury. (A–C) BAL neutrophils, macrophages, and protein; (D) pulmonary *Il17a* mRNA expression; (E–I) BAL G-CSF, IL-6, IP-10, KC, and MCP-1. Results are mean±SEM of 4–11 mice per group. *p<0.05 versus genotype-matched air-exposed mice. ^#^p<0.05 versus WT mice with the same exposure.

In WT mice, O_3_ exposure caused a time-dependent increase in BAL neutrophils, macrophages, and protein (a measure of O_3_-induced lung injury [41]) (Fig. 1A–C), consistent with previous reports by ourselves and others [18], [22], [23], [41], [42]. Increases in BAL inflammatory cells were significantly reduced in TCRδ^−/−^ versus WT mice after 48 (neutrophils) and 72 (neutrophils and macrophages) hours of exposure (Fig. 1A,B). BAL protein was also reduced in TCRδ^−/−^ versus WT mice after 72 hours exposure, but not at earlier times (Fig. 1C).

Several cytokines, including KC, IL-6, IP-10 (CXCL10), G-CSF, MCP-1 and IL-17A [17], [18], [22], [23], [41]–[44], can contribute to inflammatory cell recruitment to the lungs after O_3_ exposure. BAL IL-17A expression was below the limits of detection of ELISA. Consequently, we used q-RT-PCR to measure IL-17A. *Il17a* mRNA abundance increased after 24, 48 and 72 hours of O_3_ in WT but not TCRδ^−/−^ mice (Fig. 1D). O_3_-induced increases in BAL concentrations of BAL G-CSF, IL-6, KC and IP-10 were each reduced in TCRδ^−/−^ versus WT mice at 72 hours of exposure (Fig. 1E–H). For G-CSF and IP-10, there was a similar trend at 24 and 48 hours (Fig. 1E,G). γδ T cell deficiency had no effect on O_3_-induced changes in BAL MCP-1, although MCP-1 trended lower in TCRδ^−/−^ versus WT mice at 72 hours.

### IL-17A^+^ γδ T Cells are Increased by O_3_ Exposure

**Figure 2:**
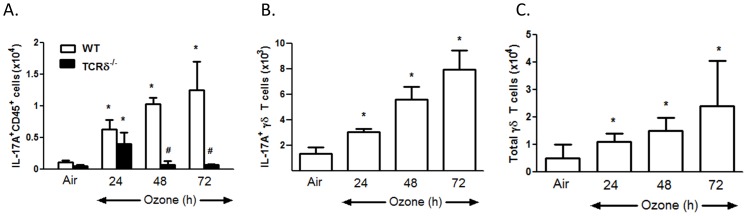
Effect of O_3_ exposure on IL-17A positive lung cells assessed by flow cytometry. (A) lung IL-17A^+^CD45^+^; (B) lung IL-17A^+^ γδ T cells; (C) total lung γδ T cells. Results are mean±SEM for 3–6 air-exposed and 4–11 O_3_-exposed mice. *p<0.05 versus genotype-matched air-exposed mice. ^#^p<0.05 versus WT mice with same exposure.

Flow cytometry indicated that the number of IL-17A^+^ CD45^+^ cells was significantly increased by O_3_ in WT mice. This effect was ablated in TCRδ^−/−^ mice (Fig. 2A). Further analysis indicated that in WT mice, the numbers of IL-17A^+^ γδ T cells as well as the total number of γδ T cells were increased by O_3_ (Fig. 2B, C), as reported previously using a similar gating strategy [18].

### Effect of Anti-IL-17A Treatment

**Figure 3:**
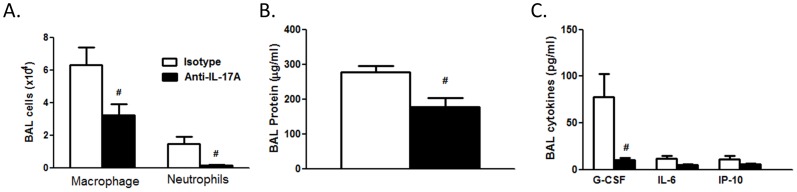
Effect of anti-IL-17A on O_3_-induced pulmonary inflammation and injury. WT mice were injected with anti-IL-17A or isotype 24 h prior to O_3_ (0.3 ppm O_3_ for 72 h). (A) BAL macrophages and neutrophils; (B) BAL protein; (C) BAL cytokines determined by multiplex assay. Results are mean±SEM of 5–7 mice per group. ^#^p<0.05 versus isotype control.

Compared to isotype control, anti-IL-17A treatment of WT mice caused a significant reduction in BAL neutrophils and macrophages (Fig. 3A). Anti-IL-17A treatment also significantly decreased BAL protein (Fig. 3B) and BAL G-CSF (Fig. 3C). Given this key role for IL-17A, these data indicate that the decreased inflammatory response observed in the TCRδ^−/−^ mice was likely due to the lack of *Il17a* expression (Fig. 1D) and demonstrate that G-CSF likely contributes to the effect of IL-17A on neutrophil recruitment.

### Role of TNFα

**Figure 4:**
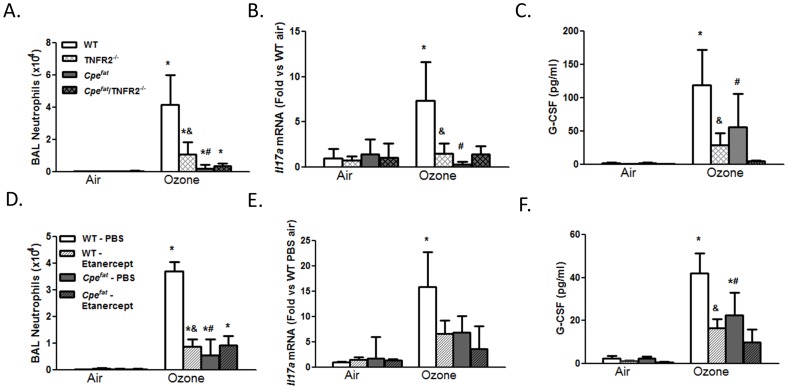
Impact of TNFR2 deficiency (A–C) or etanercept (D–F) on O_3_-induced inflammation in obese (*Cpe^fat^*) and lean (WT) mice. (A, D) BAL neutrophils; (B, E) *Il17a* mRNA expression; (C, F) BAL G-CSF. Results are mean±SE of data from 3–11 mice in each group.*p<0.05 versus air-exposed mice of same genotype and treatment; ^#^p<0.05 versus exposure matched lean mice with same TNFR2 genotype or treatment; & p<0.05 versus TNFR2 sufficient (A–C) or vehicle treated mice (D–F) with same exposure and Cpe genotype.

**Figure 5:**
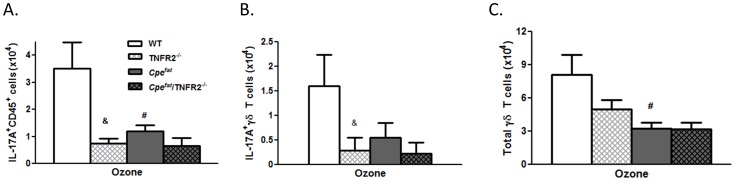
Role of TNFα for IL-17A expression in γδ T cells. Total number of (A) lung IL-17A^+^CD45^+^ cells; (B) lung IL-17A^+^ γδ T cells; and (C) total lung γδ T cells. Results are mean±SE of data from 5–6 mice in each group. ^#^p<0.05 compared to lean mice with same TNFR2 genotype; & p<0.05 compared to TNFR2^+/+^ Cpe genotype matched mice.

BAL neutrophils were significantly lower in TNFR2^−/−^ versus WT mice exposed to O_3_ for 48 h (Fig. 4A), consistent with the results of Cho et al [22]. Similar results were obtained in WT mice treated with etanercept versus vehicle (Fig. 4D). O_3_ exposure caused a significant increase in pulmonary *Il17a* expression in WT mice (Fig. 4B), consistent with results described above (Fig. 1D). However in TNFR2^−/−^ mice, no such increase in *Il17a* mRNA abundance was observed (Fig. 4B). Similar results were obtained in mice treated with etanercept (Fig. 4E). Flow cytometry also indicated a decrease in IL-17A^+^CD45^+^ cells in O_3_-exposed TNFR2^−/−^ versus WT mice (Fig. 5A). This change was due to decreased numbers of IL-17A^+^ γδ T cells (Fig. 5B). BAL G-CSF was also significantly lower in O_3_-exposed TNFR2^−/−^ versus WT mice (Fig. 4C) and in etanercept treated versus vehicle treated WT mice (Fig. 4F).

**Figure 6:**
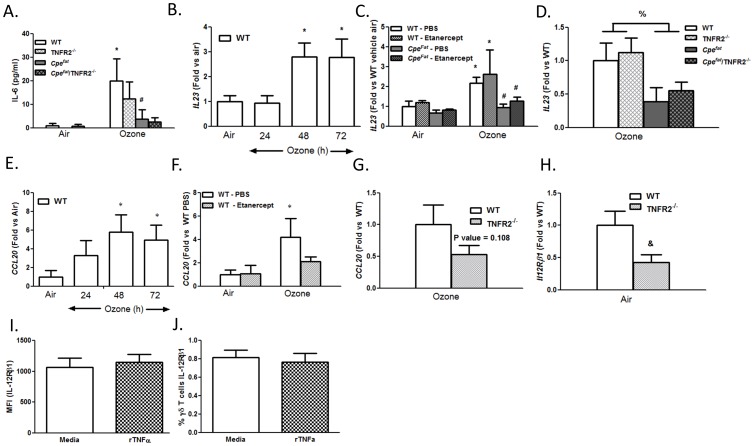
TNFα signaling is required for expression of *Il12Rβ1* and *Ccl20*. (A) BAL IL-6; (B–D)) *Il23 (p19)* mRNA; (E–G) *Ccl20* mRNA; (H) *Il12Rβ1* mRNA; (I) MFI and (J) % of γδ T cells positive for IL-12Rβ1 after stimulation with TNFα Results are mean±SE of data from 3–11 mice in each group. *p<0.05 versus air exposed mice of the same genotype; ^#^p<0.05 versus exposure matched lean mice with the same TNFR2 genotype or treatment; & p<0.05 versus WT; %<0.05 obese versus lean regardless of TNFR2 genotype.

The requirement of IL-23 and IL-6 for IL-17A expression in γδ T cells [45], [46], suggested that reductions in IL-17A^+^ γδ T cells in TNFR2^−/−^ mice might be the result of loss of TNFα-induced expression of IL-23 or IL-6. O_3_ increased BAL IL-6 in WT mice (Fig. 1F) and O_3_ also increased pulmonary *Il23* (p19) mRNA abundance (Fig. 6B), but neither IL-6 nor IL-23 were affected by TNFR2 deficiency or etanercept treatment (Fig. 6A, C). In contrast, TNFR2^−/−^ mice had reduced expression at baseline of *Il12Rβ1* (Fig. 6H), a component of the IL-23 receptor. A similar trend was observed in etanercept treated mice (data not shown). O_3_ exposure had no effect on *Il12Rβ1* (data not shown). Expression of the other component of the IL-23 receptor, *Il23R,* was not affected by TNFR2 deficiency (data not shown). To determine if TNFα was having direct effects on *Il12Rβ1*expression on γδ T cells, we isolated total lung cells from WT mice, stimulated them overnight with TNFα and examined IL-12Rβ1 expression on γδ T cells by flow cytometry (Fig. 6I,J). TNFα had no effect on the levels of IL-12Rβ1 on γδ T cells as measured by MFI and did not affect the percentage of γδ T cells expressing IL-12Rβ1, suggesting that other cells in the lung accounted for differences in *Il12Rβ1* mRNA expression.

We also considered the possibility that TNFα might impact the recruitment of γδ T cells to the lung. In WT mice, O_3_ exposure caused an increase in pulmonary mRNA expression of *Ccl20* (Fig. 6E), a chemoattractant for IL-17A^+^ cells [47], [48], whereas no such increase was observed in mice treated with etanercept (Fig. 6F), suggesting that the role of TNFα is in the CCL20 dependent recruitment of IL-17^+^ γδ T cells to the lungs. Similarly, there was a trend towards reduced *Ccl20* mRNA abundance in O_3_-exposed TNFR2^−/−^ versus WT mice (Fig. 6G), although the effect did not reach statistical significance.

### Response to O_3_ in Obese Mice

*Cpe^fat^* mice, regardless of their TNFR2 genotype or exposure, weighed almost twice as much as controls (data not shown). BAL neutrophils were significantly lower in *Cpe^fat^* versus WT mice exposed to O_3_ (Fig. 4A,D), consistent with our previous observations using this exposure regimen [34]. In contrast to the substantial reduction in BAL neutrophils observed in TNFR2^−/−^ versus WT mice, TNFR2 deficiency had no significant effect on BAL neutrophils in O_3_-exposed *Cpe^fat^* mice (Fig. 4A). Similar results were obtained in etanercept treated WT mice (Fig. 4D). Cpe genotype had no impact on the number of BAL or lung macrophages (data not shown).

*Il17a* expression was significantly lower in O_3_ exposed *Cpe^fat^* versus WT mice (Fig. 4B,E). The number of IL-17A^+^ CD45^+^ cells was also significantly lower in O_3_-exposed *Cpe^fat^* than WT mice (Fig. 5A). The total number of γδ T cells and the number of IL-17A^+^ γδ T cells was also reduced in the lungs of *Cpe^fat^* versus WT mice (Fig. 5B,C). O_3_-induced increases in BAL G-CSF were also lower in *Cpe^fat^* versus WT mice (Fig. 4C, E) consistent with the reductions in IL-17A expression. Both BAL IL-6 and pulmonary *Il23* mRNA expression were lower in *Cpe^fat^* versus WT mice (Fig. 6A, C,D). Reductions in these cytokines would be expected to reduce IL-17A expression, as observed (Fig. 4B, E). Whereas TNFR2 deficiency and etanercept reduced *Il17a* mRNA, IL-17A^+^ γδ T cells, and BAL G-CSF in lean WT mice, neither TNFR2 deficiency or etanercept affected these outcomes in obese *Cpe^fat^* mice (Fig. 4B,C and 5A–C).

## Discussion

Our data indicate a key role for IL-17A^+^ γδ T cells in the pulmonary inflammation induced by subacute O_3_. Our data also indicate that TNFα promotes pulmonary inflammation after subacute O_3_ by inducing recruitment of IL-17A^+^ γδ T cells, likely via *Ccl20* expression. Finally, our data suggest that the attenuated pulmonary inflammation observed in obese mice after subacute O_3_ is the result of reduced pulmonary IL-17A^+^ γδ T cells, consequent to reduced IL-23 and IL-6 expression.

Inflammatory cell recruitment to the lungs after subacute O_3_ exposure required γδ T cells (Fig. 1A,B). γδ T cells have also been shown to be required for the pulmonary inflammation observed 24 but not 8 hours after acute exposure to much higher O_3_ concentrations (2 ppm) [49], [50], consistent with the time needed for recruitment and activation of γδ T cells. However, in those studies, the precise role of these γδ T cells was not assessed. Our data indicate that after exposure to lower concentrations of O_3_ for much longer periods of time, the role of γδ T cells involved IL-17A expression. Both lung *Il17a* mRNA and lung IL-17A^+^ γδ T cells increased after subacute O_3_ exposure with a time course similar to that of neutrophil recruitment (Figs. 1A, 1D, 2B). Furthermore, O_3_-induced increases in *Il17a* mRNA abundance were abolished in TCRδ^−/−^ mice (Fig. 1D). In addition, both BAL neutrophils and macrophages were reduced in mice treated with anti-IL-17A versus isotype control antibody (Fig. 3A). This ability of IL-17A^+^ γδ T cells to control the influx of macrophages and neutrophils is consistent with the findings in other models of lung infection and injury [4], [51]–[54]. While our data indicate that IL-17^+^ γδ T cells are *required* for O_3_-induced inflammatory cell recruitment, they are not *sufficient*. For example, O_3_ is highly reactive and macrophages and epithelial cells are the initial targets of its action. These cells are the likely source of TNFα which is required for neutrophil recruitment (Fig. 4) perhaps via induction of CCL20 and consequent recruitment IL-17A+ γδ T cells (Figs. 5,6). Epithelial cells are also the likely source of CCL20. Furthermore, macrophages also produce IL-17A after O_3_ exposure [18], and the role of γδ T cells may be to promote these effects. Macrophages and epithelial cells are also the likely source of other chemokines that interact with IL-17A (see below) to promote neutrophil recruitment.

IL-17A has direct chemoattractant effects on macrophages [55], which likely explains the ability of anti-IL-17A to attenuate O_3_-induced increases in BAL macrophages (Fig. 3A). In contrast, IL-17A induces neutrophil recruitment to the lungs by inducing expression of other neutrophil chemotactic and survival factors. With subacute O_3_ exposure, G-CSF appears to be one of these factors. In WT mice, the time courses of induction of BAL G-CSF and *Il17a* expression were similar (Fig. 1D,E). Importantly, anti-IL-17A and γδ T cell deficiency each caused a marked and significant reduction in BAL G-CSF in O_3_ exposed mice (Fig. 1E, 3C). The data are also consistent with our previous observations showing reductions in BAL G-CSF in O_3_-exposed adiponectin-deficient mice treated with anti-IL-17A [18]. The observed role of IL-17A in G-CSF expression is in agreement with previous reports indicating that IL-17A signaling increases the transcription and stability of the *Gcsf* mRNA [56], [57], via effects on ERK1/2 activation [58]. G-CSF causes neutrophil release from bone marrow and promotes neutrophil survival [59]. Since serum G-CSF did not increase after subacute O_3_ exposure (data not shown), G-CSF is unlikely to act via effects on bone marrow in this model. Instead, G-CSF likely contributes by increasing the survival of neutrophils recruited to the lungs in response to other factors such as IP-10 (Fig. 1G).

TNFα is not directly chemotactic for neutrophils [24]. However, in lean WT mice, TNFR2 deficiency or the TNFα antagonist, etanercept, reduced the O_3_-induced increase in BAL neutrophils (Fig. 4A,D) consistent with previous reports [22], [23], [60] indicating a role for TNFα in neutrophil recruitment induced by subacute O_3_. TNFα also contributes to neutrophil recruitment in other conditions (reviewed in [61]), though the mechanism is not well understood. Our data suggest that at least in the setting of O_3_ exposure, the ability of TNFα to recruit neutrophils involves IL-17A and that the source of this IL-17A is γδ T cells (Fig. 5). O_3_-induced increases in pulmonary *Il17a* expression were attenuated in TNFR2^−/−^ versus WT mice (Fig. 4B) and in etanercept versus vehicle treated WT mice (Fig. 4E). The number of IL-17A^+^ γδ T cells in the lung was also lower in TNFR2^−/−^ versus WT mice exposed to O_3_ (Fig. 5A,B). The ability of TNFα to promote pulmonary IL-17A expression after O_3_ exposure is consistent with the role of TNFα in other pathogenic states. For example, etanercept reduces the elevated blood and skin Th17 cells observed in patients with psoriasis [26]. Similarly, another anti-TNFα therapy, infliximab, reduces IL-17A in ocular fluid from uveitis patients with Behcet’s disease [27].

To better understand the role of TNFα, we examined IL-6 and IL-23 expression. Both these cytokines can contribute to induction of IL-17A in γδ T cells [45], [62]. Both IL-6 and IL-23 were induced in the lungs after O_3_ exposure, but were not affected by TNFR2 deficiency or by etanercept (Fig. 6A,C,D), indicating that TNFα is not required for their expression. We did observe that mRNA expression of one of the two subunits of the IL-23 receptor, *Il12Rβ1*, was decreased (Fig. 6H) in unexposed lungs from TNFR2^−/−^ mice. Similar trends were observe after etanercept treatment (data not shown). Since others have reported that TNFα can act directly on γδ T cells [40], [63], we considered the possibility that TNFα was acting to increase *Il12Rβ1* expression on γδ T cells, thus increasing their ability to respond to IL-23. However, culture of lung cells with TNFα resulted in no change in surface bound IL-12Rβ1 on γδ T cells (Fig. 6I,J). Instead, our data, suggest that effects of TNFα on *Ccl20* expression (Fig. 6F,G) account for the observed effects of TNFα/TNFR blockade on IL-17A^+^ γδ T cells. Ccl20 acts via CCR6, a receptor expressed by IL-17A^+^ γδ T cells that promotes chemotaxis of these cells [64]. TNFα is also required for pulmonary Ccl20 expression after acute O_3_ exposure (2 ppm for 3 h) [37]. A role for TNFα in *Ccl*20 expression has also been demonstrated in dermal lesions of psoriasis patients based on treatment with the TNFα antagonist infliximab [65].

We observed fewer neutrophils in BAL fluid of obese *Cpe^fat^* versus lean WT mice after subacute O_3_ exposure (Fig. 4A,D), consistent with previous observations [34]. Reduced responses are observed in *Cpe^fat^* mice not only after 48 h exposure (Fig. 4A,D), but also after 24 or 72 h exposures [34]. Pulmonary *Il17a* expression and IL-17A^+^ γδ T cells were also reduced in the obese mice, as was the total number of γδ T cells (Fig. 4). BAL G-CSF was also lower in *Cpe^fat^* versus lean WT mice (Fig. 4C,F). Moreover, O_3_-induced increases in BAL IL-6 and pulmonary *Il23* expression were also reduced in *Cpe^fat^* versus WT mice (Fig. 6C,D). TNFR2 deficiency or etancercept treatment in *Cpe^fat^* mice did not further reduce BAL neutrophils or pulmonary *Il17a* expression, in contrast to what was observed in WT mice (Fig. 4B,E). Given the already reduced numbers of total γδ T cells in *Cpe^fat^* mice exposed to O_3_ (Fig. 5C), and our observations indicating the key role for IL-17A^+^ γδ T cells in the effects of TNFα on neutrophil recruitment, it is not surprising that TNFα had no further effect on the response to O_3_ in obese mice. Taken together, the data suggest that obesity-related reductions in neutrophil recruitment induced by subacute O_3_ exposure are the result of reduced IL-17A-dependent G-CSF release, consequent to reduced IL-6 and IL-23 expression. However, we cannot rule out the possibility that other factors also contributed. For example, neutrophils from obese mice exhibit reduced chemotactic activity towards CXCR2 ligands [66]. Such defects in neutrophil chemotaxis would also be expected to reduce O_3_-induced neutrophil recruitment in *Cpe^fat^* mice.

In addition to affecting responses to O_3_, obesity also impacts responses to bacterial and viral infections [67]–[71]. As described above, IL-17^+^ γδ T cells contribute to neutrophil recruitment and pathogen clearance after certain bacterial infections [3], [4]. IL-17^+^ γδ T cells are also required for clearance of secondary infections after influenza [72]. Hence, obesity-related changes in IL-17^+^ γδ T cells (Figs. 4b, 5a,b) may contribute not only to obesity-related alterations in responses to O_3_, but may have broader implications for effects of obesity on host defense. In support of this, obese mice compared to lean mice have fewer skin γδ T cells number and the few γδ T cells they have are dysfunctional [73], which leads to impairment in wound healing. These decreases in γδ T cells numbers and impairment in function of the skin in obese mice are due to altered STAT5 signaling and chronic TNFα signaling [74].

In summary, our data indicate that γδ T cells are required for the pulmonary inflammation that occurs after subacute O_3_ exposure in mice via their ability to produce IL-17A. IL-17A then leads to G-CSF expression. Our data also indicate that TNFα is required for recruitment IL-17A^+^ γδ T cells to the lungs likely through its ability to induce *Ccl20*. These results emphasize the importance of γδ T cells not only for pathogen clearance, but also for responses to other insults that induce oxidative stress, and describe a new role for TNFα in these events. Finally, our data indicate that obesity-related reductions in the ability of subacute O_3_ to promote neutrophil recruitment to the lungs are the result of reduced IL-17A^+^ γδ T cells. These results suggest that other conditions that impact γδ T cell recruitment or activation will also impact responses to this common pollutant.
